# Effect of High Sugar Intake on Glucose Transporter and Weight Regulating Hormones in Mice and Humans

**DOI:** 10.1371/journal.pone.0101702

**Published:** 2014-07-10

**Authors:** Yvonne Ritze, Gyöngyi Bárdos, Jan G. D’Haese, Barbara Ernst, Martin Thurnheer, Bernd Schultes, Stephan C. Bischoff

**Affiliations:** 1 Department of Nutritional Medicine, University of Hohenheim, Stuttgart, Germany; 2 Department of Surgery, Klinikum Rechts der Isar, Technische Universität München, Munich, Germany; 3 Interdisciplinary Obesity Center, Rorschach, Switzerland; INRA, France

## Abstract

**Objective:**

Sugar consumption has increased dramatically over the last decades in Western societies. Especially the intake of sugar-sweetened beverages seems to be a major risk for the development of obesity. Thus, we compared liquid versus solid high-sugar diets with regard to dietary intake, intestinal uptake and metabolic parameters in mice and partly in humans.

**Methods:**

Five iso-caloric diets, enriched with liquid (in water 30% vol/vol) or solid (in diet 65% g/g) fructose or sucrose or a control diet were fed for eight weeks to C57bl/6 mice. Sugar, liquid and caloric intake, small intestinal sugar transporters (GLUT2/5) and weight regulating hormone mRNA expression, as well as hepatic fat accumulation were measured. In obese versus lean humans that underwent either bariatric surgery or small bowel resection, we analyzed small intestinal GLUT2, GLUT5, and cholecystokinin expression.

**Results:**

In mice, the liquid high-sucrose diet caused an enhancement of total caloric intake compared to the solid high-sucrose diet and the control diet. In addition, the liquid high-sucrose diet increased expression of GLUT2, GLUT5, and cholecystokinin expression in the ileum (P<0.001). Enhanced liver triglyceride accumulation was observed in mice being fed the liquid high-sucrose or -fructose, and the solid high-sucrose diet compared to controls. In obese, GLUT2 and GLUT5 mRNA expression was enhanced in comparison to lean individuals.

**Conclusions:**

We show that the form of sugar intake (liquid versus solid) is presumably more important than the type of sugar, with regard to feeding behavior, intestinal sugar uptake and liver fat accumulation in mice. Interestingly, in obese individuals, an intestinal sugar transporter modulation also occurred when compared to lean individuals.

## Introduction

Sugar consumption has increased dramatically over the last decades in Western societies and is regarded as a major risk for the development of obesity [1]. Particularly, changes in dietary and eating behavior such as preferring sugar-sweetened beverages and sugar-rich processed food, in addition to a sedentary life style, are associated with the sharp rise in obesity [2]–[4]. Among the dietetic factors, sucrose- and fructose-rich soft drinks typically consumed in addition to meals are leading to enhanced energy uptake and emerge as the most consistent factor causing obesity [5]–[8].

Furthermore, a probable link exists between dietary fructose intake and obesity-associated diseases such as non-alcoholic fatty liver disease (NAFLD) and insulin resistance in humans [6]–[9].

The hypothesis to be tested is, if alterations of intestinal sugar-uptake and -signaling contribute to the development of obesity. Indeed, various studies have shown that altered sugar signaling pathways influence feeding behavior, modulate intestinal sweet taste receptors, sugar transporters, and alter weight-regulating gastrointestinal hormone expression [10]–[13]. Short-term studies in mouse and man revealed that monosaccharide transport across epithelial membranes in the intestine is mediated by the family of sodium-driven sugar co-transporters (SGLTs) and glucose transporters (GLUTs), respectively [14]. SGLT1, is a low-capacity, high-affinity transporter and the only transporter capable of moving glucose against a concentration gradient. While SGLT1 is saturated already at millimolar glucose levels, facilitated diffusion via GLUT2 seems to be the principal route for glucose and fructose absorption [15]. GLUT2 is the glucose transporter with the lowest affinity/specificity and the highest capacity for glucose (15). In addition, GLUT2 is capable of recognizing galactose and has been involved in the control of food intake in the hypothalamus [16].

GLUT5 is the only low-affinity high-capacity transporter specific and essential for the fructose uptake with no ability to transport glucose or galactose [17], [18]. Intestinal GLUT5 expression might be affected in the course of obesity and metabolic diseases. Nevertheless, data revealing the role of GLUT5 and GLUT2 in causing, contributing to or exacerbating the above mentioned diseases remain controversial [18], [19].

Sugars might also alter metabolism by modulating enteroendocrine cells. Enteroendocrine cells are known to act as primary chemoreceptors, sources of gastrointestinal hormones and peptides. Indirect evidence suggests a connection between sugar absorption and the secretion and function of some peptides [12], [20]–[22]. Thus, enhanced sugar uptake observed in obesity can augment energy uptake, but also alter sugar transport across the brush border membrane and gastrointestinal hormone release in the intestine.

Many questions remain open about the role of sugars, namely sucrose and fructose, in obesity and associated diseases. Most studies in the field are restricted to short-term effects of sugars on energy metabolism and other parameters. In the majority of cases the study designs are variable, and type (e.g. fructose versus glucose) and texture (liquid versus solid form) of the sugars are hardly analyzed in detail [7], [8], [23].

The aim of the present study was to investigate the influence of type and texture of dietetic sugars in mice. We fed liquid and solid high-fructose and -sucrose diets, and analyzed their influence on feeding behavior as well as the development of obesity and fatty liver disease. In obese and lean humans, intestinal sugar transporter and weight regulating hormone expression was analyzed.

## Materials and Methods

### Mice and treatments

Mice were housed in ‘Individually Ventilated Cages’ (IVCs) with cedar bedding in a pathogen-free barrier facility accredited by the Association for Assessment and Accreditation for Laboratory Animal Care International (AAALAC). All procedures were approved by the local Institutional Animal Care and Use Committee (Regional Council Stuttgart, permit number: V 265/09 EM).

**Figure 1:**
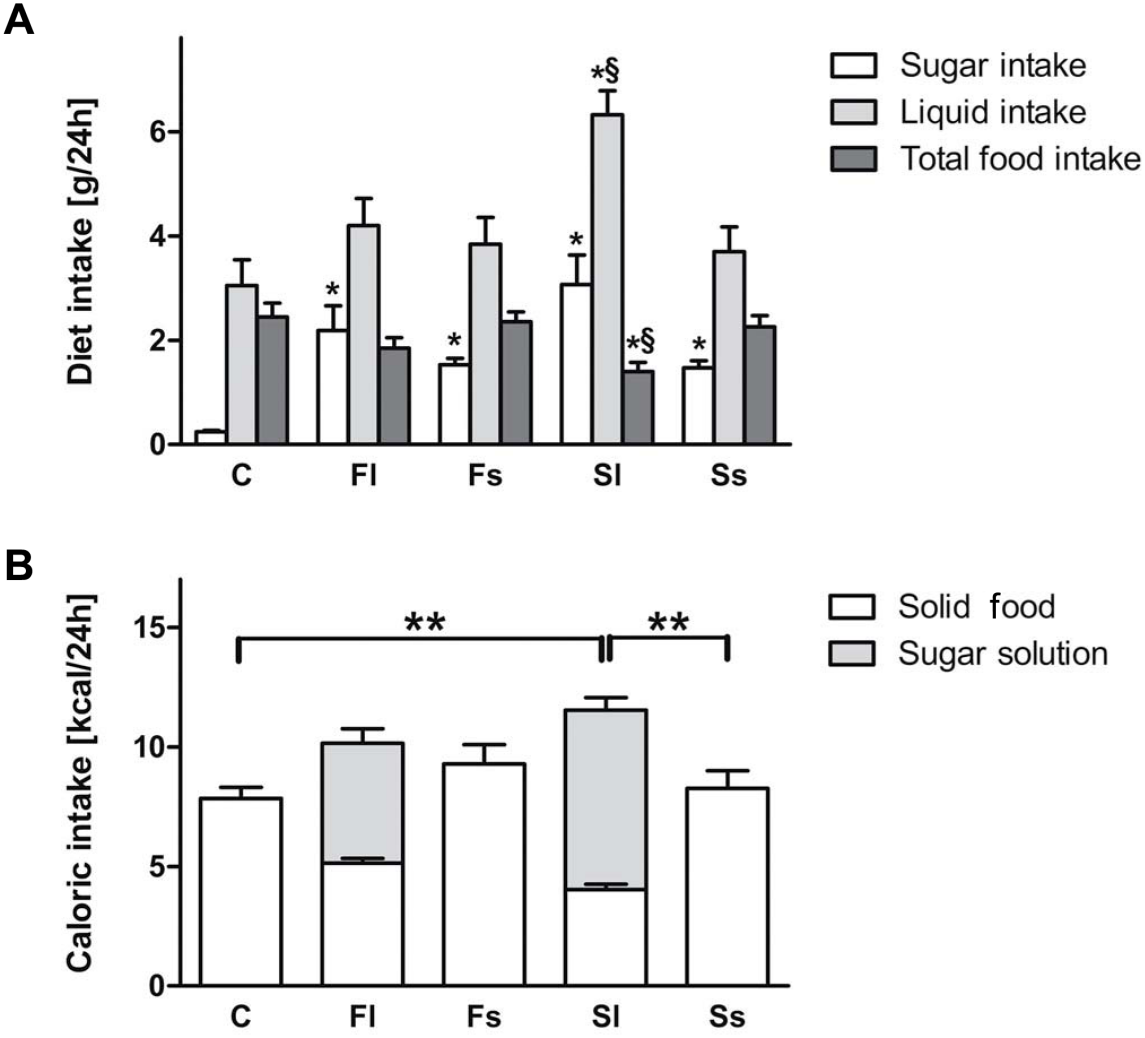
Elevated liquid sucrose intake increased caloric intake in mice. Sugar, liquid and food intake was analyzed (A). Total caloric, liquid and food caloric intake was determined (B). Data in panels A and B are shown as means ± SEM (n = 10). */**P<0.01, compared to control; ^§^P<0.05, compared to sucrose solid. C, control; Fl, fructose liquid; Fs, fructose solid; Sl, sucrose liquid; Ss, sucrose solid.

In two independent experiments, we investigated 6 weeks old female C57BL/6 mice (Janvier, Saint Berthevin Cedex, France). The mice were divided into five groups (n = 10 per group) according to five different dietetic regimes provided ad libitum over eight weeks. Group 1 (controls, C) received water and mouse breeding (MZ)-diet (standard diet from Sniff, Soest, Germany) containing 10% (g/g) sugars. Groups 2 (fructose liquid, Fl) and 3 (sucrose liquid, Sl) received water supplemented with fructose or sucrose at 30% (vol/vol), respectively, and enriched MZ-diet to compensate for reduced food uptake. Groups 4 (fructose solid, Fs) and 5 (sucrose solid, Ss) received water and the high-fructose or -sucrose diet containing 65% (g/g) sugars, which equals the sugar amount per day that mice ingested when offered sugar water at 30%. This approach resulted in a similar sugar uptake of about 2 g/d among all the groups except the control group (Figure 1A).

Every two weeks the mice were placed in metabolic cages for 24 h, to which they were acclimatized to before. Measuring food and liquid intake g/d in the metabolic cages, the sugar and caloric intake for each mouse was calculated.

After 8 weeks, the mice were weighted and anesthetized via intra peritoneal administration (ketamine at 80 mg/kg and xylazin at 6 mg/kg body weight). Blood was collected from the portal vein prior to euthanatizing and plasma glucose in non-fasted mice was measured (Laboratory analysis, Sindelfingen, Germany). Specimen of duodenum and liver tissue were frozen immediately in liquid nitrogen for RNA and protein extraction. Portions of liver tissue were snap-frozen and fixed in Tissue Tek O.C.T. compound (Sakura Finetek Europe, AV Alphen aan den Rijn, Netherlands) for subsequent sectioning and mounting on microscope slides.

### Hepatic lipid analysis

Liver tissue pieces (50–100 mg) were homogenized in ice-cold 2x PBS and lipids were extracted. Triglycerides were assessed by chemo-luminescence using a commercial kit (Randox, Krefeld, Germany). Values were normalized to protein concentration, determined by Bradford assay, in liver homogenates (Bio-Rad Laboratories, Munich, Germany).

To determine hepatic lipid accumulation, frozen sections of liver (10 µm) were stained with Oil Red O and counterstained with hematoxylin (Sigma, Steinheim, Germany). Representative photomicrographs were captured at a 400x magnification using Axio Vert 200 M (Zeiss, Jena, Germany).

### Endotoxin assay

Portal plasma samples were heated at 72°C for 20 min. Endotoxin concentration was determined using a limulus amebocyte lysate assay kinetic kit (concentration range 0.015–1.2 EU/mL; Charles River, Wilmington, MA). To minimize analysis errors, samples were spiked.

### RNA isolation and real-time RT-PCR

Total RNA was extracted from murine ileum or human jejunal tissue samples using TriFast reagent (PEQLAB, Erlangen, Germany). RNA concentrations were determined by spectrophotometry, before 0.25 µg total RNA was reverse transcribed with an iScript DNA synthesis kit (Bio-Rad Laboratories, Munich, Germany) followed by a DNAse digestion step (Fermentas, St. Leon Rot, Germany). PCR primers were designed using Primer3 software (Whitehead Institute for biomedical research, Cambridge, MA, USA) (Table 1). SsoFast EvaGreen Supermix (Bio-Rad Laboratories, Munich, Germany) was used to prepare the PCR mix. The amplification reactions were carried out in an iCycler (Bio-Rad Laboratories, Munich, Germany) with 40 cycles of a two-step PCR (denaturation 95°C for 35 s, denaturation 95°C for 5 s, annealing/extension 62°C for 10 s). The fluorescence intensity of each sample was measured at each temperature change to monitor amplification of the target gene. The comparative CT-method was used to determine the amount of target gene, normalized to an endogenous reference gene (18S) and relative to a calibrator (2^−ΔΔCt^). The purity of PCR products was verified by melting curves and gel electrophoresis.

**Table 1:** Primers used for mRNA detection.

	Forward (5′-3′)	Reverse (5′-3′)
**GLUT2**	TGCACATGGCCCAGCAGTTCT	GCAGCACAGAGACAGCCGTGAA
**hGLUT2**	CTCTCCTTGCTCCTCCTCCT	TTGGGAGTCCTGTCAATTCC
**GLUT5**	ACCTCAGCGCAGGCGTGAAA	AGCAGGCTATGAGGCAGGTGGA
**hGLUT5**	ATCTCCGTGCTGAAGCTGTT	GCGCTCAGGTAGATCTGGTC
**SGLT1**	ACTGCCACCGATGCACCCAT	AAACATGGCCCACAGCCCGA
**T1R3**	ACCCGGAGCGCAACACTTCA	ACAAGGAACACCGGGAGCGT
**CCK**	GCCGAGGACTACGAATAC	GCATAGCAACATTAGGTCTG
**hCCK**	CAGAGGAGGCAGAATAAGAA	CAGGAGTCACAGATGAAGAA
**Ghrelin**	ATCTGTCCTCACCACCAA	GCTCCTCCTCTGTCTCTT
**Nesfatin-1**	ACAAAATGCAGAGGACGATA	CTAGGTGAATAACTGTTGCT
**PYY**	ACTACACCGACTTCACTTG	GGACAGGGAAATGAACACA
**18S**	ATCAGATACCGTCGTAGTTC	CCAGAGTCTCGTTCGTTAT

GLUT2/5, glucose transporter 2/5; h, human; SGLT1, sodium-driven sugar co-transporter 1; T1R3, taste receptor type 1 member 3; CCK, cholecystokinin; PYY, Peptide YY.

### Human samples

Whole jejunal tissue from 20 obese (BMI = 43.1±6.0 kg/m^2^) who underwent bariatric surgery (Roux-en-Y gastric bypass, Hospital St. Gallen, CH) and 14 lean patients (BMI 24.8±3.5 kg/m^2^) who underwent gut surgery for different reasons (e.g. cancer, Hospital Rechts der Isar, Munich) were analyzed. All patients gave written informed consent to the study, which was approved by the local ethics committees (permit number Stuttgart, Germany: 87/2009 BO1; Munich, Germany: 1926/07; Rorschach, Switzerland: EKSG 10/024/2B).

### Calculation of metabolisable energy

The metabolisable energy is the difference between gross energy in consumed food (determined by bomb calorimetry) and energy in feces and urine (also measured by bomb calorimetry). The literature shows examples of macronutrients from which the heat of combustion and/or the coefficient of availability was measured [24]. With this outcome one may calculate the available energy (Table S1) from the macronutrients used in our diets. We corrected the macronutrient protein for urine extraction, as suggested by Rubner and Atwater (Table S1) [25]–[27].

### Statistical analyses

All results are presented as means ± SEM. Results from different groups were compared by one-way ANOVA, or by the Kruskal Wallis test if variances calculated with Bartlett’s test varied significantly. If significant differences between groups occurred we used the Tukey’s post-hoc test to identify the particular groups that caused the differences. In addition, a two-way ANOVA was used to understand interactions between the form (liquid versus solid) and the type of sugar (fructose versus sucrose) (Table 2). An error value of P<0.05 was defined as the level of significance prior to study start. The software GraphPad Prism 5 (GraphPad Software, La Jolla, CA) was used for calculations and graph design.

**Table 2:** Comparison of liquid and solid high-sugar diets as well as sugar type.

	Fl	Fs	Sl	Ss
**GLUT2**	250±86.2***	20.0±7.7	454±137***	31.4±12.4
**CCK**	10.6±2.5***	3.29±1.1	10.9±2.4***	1.84±0.2
**GLUT5**	18.6±3.4***	8.70±2.8^§^	37.4±6.6***	8.3±1.7^§^
**Ghrelin**	7.76±1.2	2.88±0.5^§^	4.95±0.9	10.7±1.8^§^
**TG [mol/l]**	0.30±0.03**	0.18±0.02^§§§^	0.56±0.1**	0.36±0.1^§§§^
**Liver to body ratio [g]**	6.12±0.2	6.12±0.2	5.92±0.2	6.02±0.1
**Endotoxin [EU/ml]**	0.28±0.1	0.32±0.1	0.10±0.05	0.07±0.02
**Kcal intake [kcal/24 h]**	10.1±0.5**	9.29±0.8	11.5±0.6**	8.27±0.7
**Sugar intake [g/24 h]**	2.19±0.5**	1.53±0.1	3.07±0.6**	1.47±0.1
**Liquid intake [ml/24 h]**	4.20±0.5**	3.84±0.5	6.33±0.5**	3.70±0.5
**Total food intake [g/24 h]**	1.85±0.2**	2.35±0.2	1.40±0.2**	2.26±0.2
**Ratio kcal/lean body mass [g]**	31.2±2.6*	28.9±3.5	35.0±2.9*	24.8±2.9
**Ratio kcal/body weight after dietary period [g]**	26.7±1.6*	24.3±2.4	28.2±1.7*	20.3±2.1

Liquid compared to solid sugar form (*P<0.05; **P<0.01; ***P<0.001) or fructose compared to sucrose (^§^P<0.05; ^§§§^P<0.001). Detailed feeding protocols of the four animal groups are described in material and methods. Data are means ± SEM (n = 9–10). P-values are calculated using the 2-way ANOVA. GLUT2/5, glucose transporter 2/5; CCK, cholecystokinin; TG, triglycerides; C, control diet; Fl, fructose liquid; Fs, fructose solid; Sl, sucrose liquid; Ss, sucrose solid.

## Results

### Effect of high-sugar diets on nutritional and weight parameters

Mice fed a high-sugar diet had a significant higher sugar intake compared to mice fed a control diet independent of the type and dosage form of the sugar (P<0.05; Figure 1A). We found a significant difference between the liquid and solid form of sugars analyzing kcal, sugar, liquid and food intake (P<0.01; Figure 1A), but not between the type of sugar (fructose versus sucrose). In this report, only mice fed the liquid high-sucrose diet had a significant elevated liquid intake and a reduced solid food intake compared to control mice and mice fed the solid high-sucrose diet (P<0.05; Figure 1A). Here, it is of note, that the metabolisable energy absorbed by the mice was not significant different between the liquid or solid high-sugar diets, meaning that these diets are comparable (Table S1).

Similarly, the changes in diet composition did not alter the total energy intake except in the group of mice receiving the liquid high-sucrose diet in which an enhanced energy intake was observed compared to the other groups (P<0.05; Figure 1B). Hence, the most pronounced enhancement of body weight was found in the group of mice receiving the liquid high-sucrose diet (P<0.01). Nevertheless, some weight gain was also observed in the group of mice receiving the solid high-sucrose diet (P<0.05; Table 3).

**Table 3:** Weight parameters and blood glucose.

	C	Fl	Fs	Sl	Ss
**Weight gain [g]**	1.9±0.6	2.7±0.4	2.9±0.5	4.5±0.5**	3.8±0.3*
**Liver weight [g]**	1.1±0.1	1.3±0.1	1.4±0.1**	1.3±0.1	1.4±0.1**
**Blood glucose [mg/dl]**	172.6±20.9	275.7±18.2*	282.8±14.3**	257.8±16.0*	263.3±25.4*
**Ratio kcal uptake to lean** **body mass [g]**	23.6±2.4	31.2±2.6	28.9±3.5	35.0±2.9*	24.8±2.9
**Ratio kcal uptake to body** **weight after the dietary** **period [g]**	20.9±1.5	26.7±1.6	24.4±2.4	28.2±1.7	20.3±2.1§

Data are means ± SEM (*n* = 10).

*P<0.05 and **P<0.01 compared to C;

§ P<0.05 compared to Sl.

C, control diet; Fl, fructose liquid; Fs, fructose solid; Sl, sucrose liquid; Ss, sucrose solid.

Furthermore, we calculated the ratio of kcal absorbed to grams of lean body mass and grams of adipose mass added over the dietary period. For our calculations we used the weight of the lean mice and the weight of the mice after the feeding period, since the adipose mass added is related to the lean body mass of the mice (Table 3). We found that the ratio of kcal absorbed to grams of lean body mass showed a significant difference between control and liquid sucrose fed mice. The ratio of kcal absorbed to grams of adipose mass added over the dietary period was significant difference between the group that became liquid sucrose and the group which was fed solid sucrose. Comparing the liquid and solid sugar groups and the sugar types (fructose versus sucrose) we found a significant difference between the liquid and solid sugars (P<0.05; data not shown) but not the sugar types.

The four high sugar-diets caused an increase in blood glucose and in tendency some increase in liver weight, which was more pronounced if the sugars were administered in solid form (Table 3).

### Regulation of sugar transporters and hormones in the intestine by dietetic sugars

**Figure 2:**
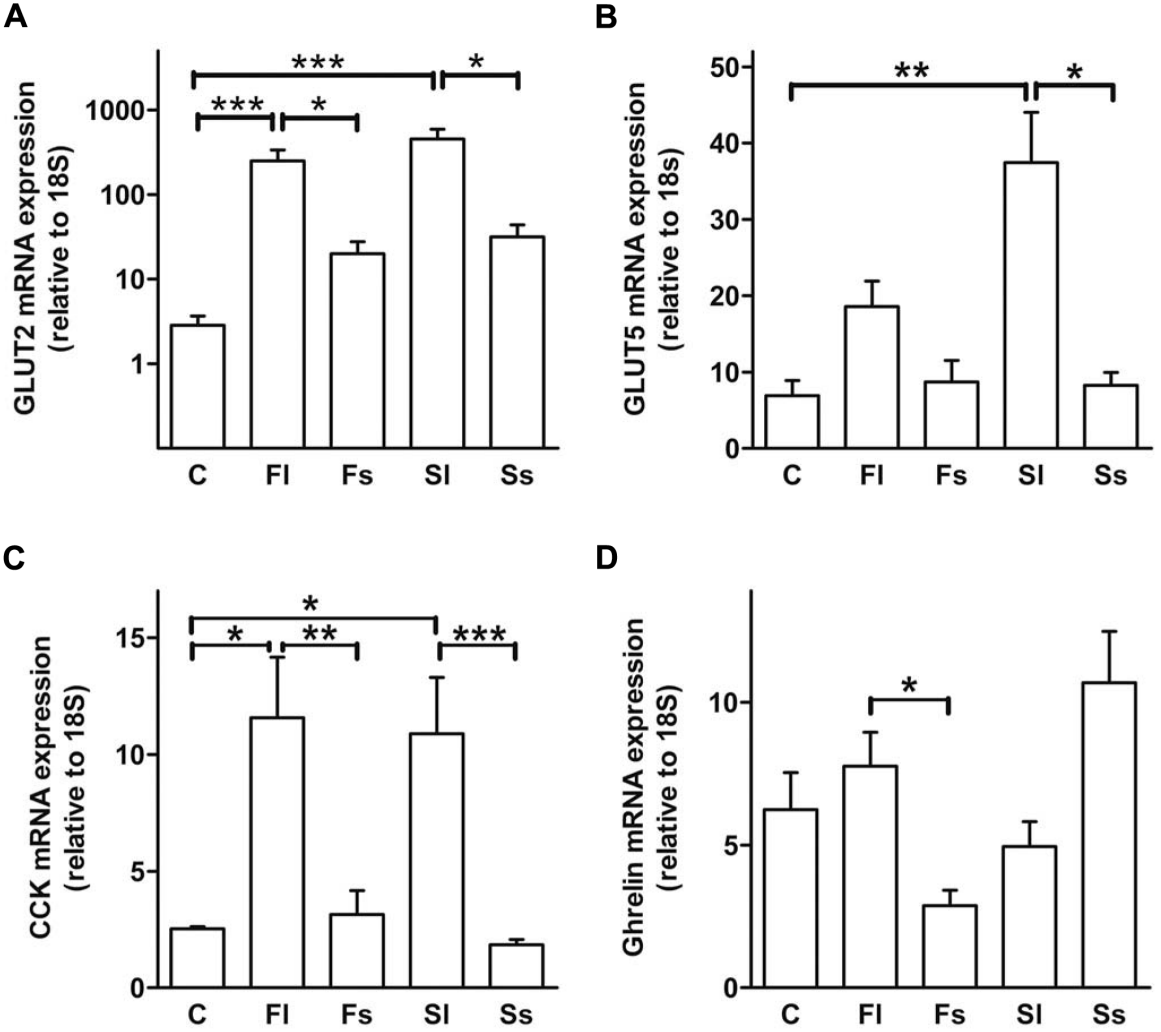
Liquid high-sugar diets increased intestinal sugar transporter and weight regulating hormone expression. Ileal GLUT2, GLUT5, CCK and ghrelin mRNA expression was detected (A/B/C/D). Data are shown as means ± SEM (*P<0.05, **P<0.01, ***P<0.001; n = 9–10). GLUT2/5, glucose transporter 2/5; CCK, cholecystokinin, for diet abbreviations see Figure 1.

Feeding high-sugar diets, we found a strongly increased GLUT2 mRNA expression (Fl = about 90 fold; P<0.001; Sl = about 160 fold; P<0.001) when sugars were dissolved in drinking water compared to the control mice. If the sugars were administered in solid form, we also observed a significant, but clearly less pronounced up-regulation of ileal GLUT2 mRNA expression (P<0.05) compared to the control mice (Figure 2A). Similar results were obtained for GLUT5 mRNA expression (Figure 2B). Comparing sugar form and type we showed a significant difference between liquid and solid sugar form for GLUT2 and GLUT5 (P<0.001) as well as a significant difference of sugar type (fructose versus sucrose) for GLUT5 (P<0.05; Table 3) within the different dietetic groups.

Both, the liquid high-fructose and -sucrose diets increased (P<0.05; P<0.01) CCK mRNA compared to solid high-sugar diets and the control diet (Figure 2C). In addition, the sugar form significantly influenced CCK (P<0.001) in contrast to the type of sugar (Table 2).

Similarly, ghrelin mRNA was slightly up-regulated feeding the fructose liquid diet compared to the fructose solid diet (P<0.05; Figure 2D). Comparing sugar form and type, we saw a significant difference between the two sugar types (P<0.05) for ghrelin expression but not the sugar forms. Nesfatin-1 mRNA was down-regulated in mice fed the sucrose liquid diet compared to the sucrose solid diet (P<0.05, data not shown).

### High-sugar diets increase hepatic lipid accumulation

**Figure 3:**
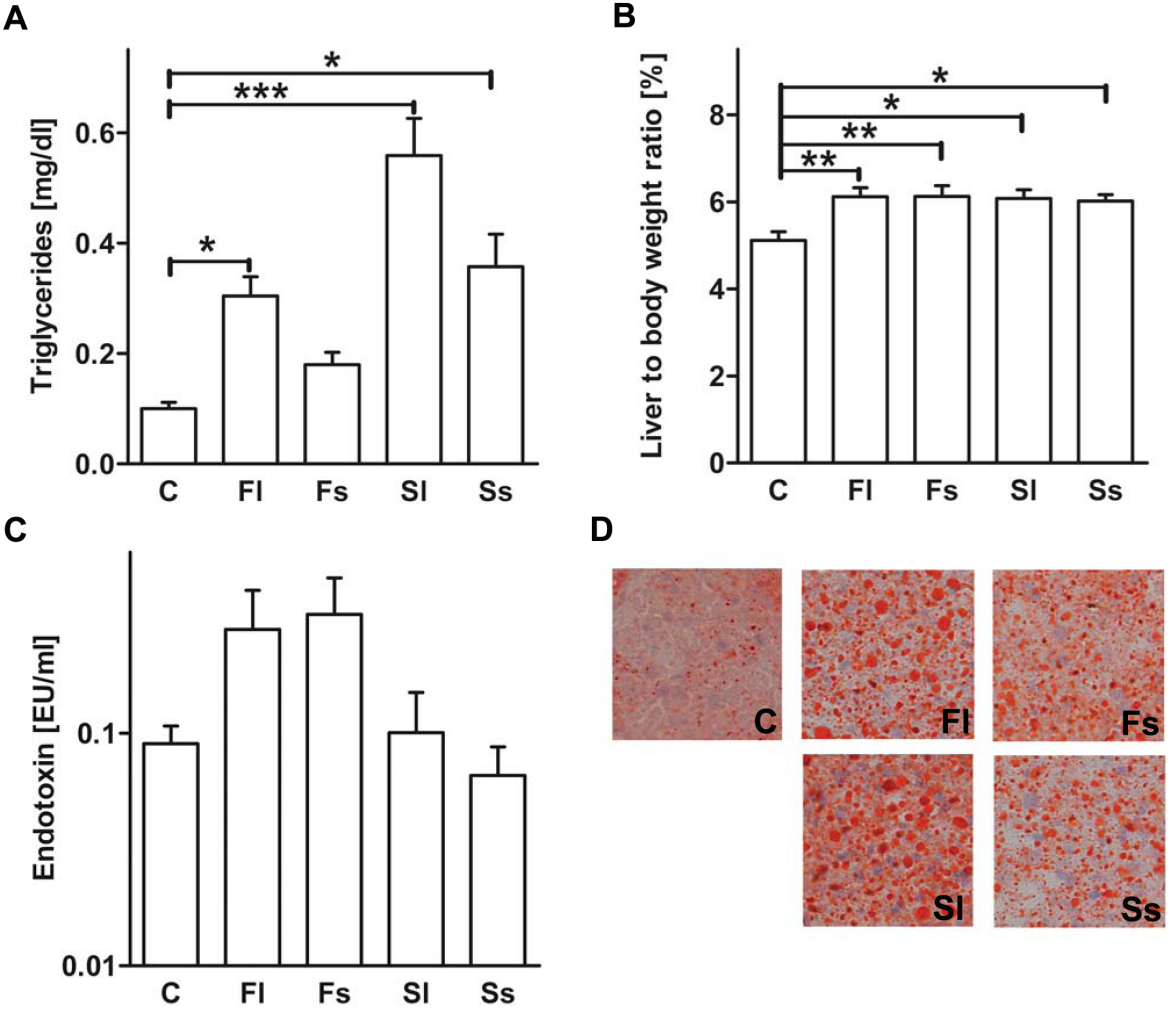
Effect of high-sugar diets on hepatic lipid accumulation. Concentrations of triglycerides in the liver (A), and liver to body ratio (B) were detected. Portal endotoxin (C), and Oil Red O staining showing fat accumulation in the liver (D) are shown. Data is shown as means ± SEM (*P<0.05, **P<0.01, ***P<0.001; n = 6–10). For Abbreviations see Figure 1.

Hepatic fat accumulation was investigated depending on whether high-sugar diets or sugars in liquid or solid form were administered to the mice. Hepatic triglycerides were increased (P<0.05) in mice receiving high-sugar diets, except in mice being fed the liquid high-fructose diet, in comparison to the control mice (Figure 3A). Similarly, the liver to body weight ratio was significantly enhanced in all the high-sugar diet fed mice compared to the control mice (Figure 3B). A noticeable but not significant increase in portal endotoxin levels following consumption of the high-fructose diets, was measured (Figure 3C). The sugar form and type significantly influenced triglycerides but not liver to body ratio or endotoxin concentration (Table 2). Overall fatty acid accumulation in the liver seemed to be more pronounced in the liquid high-sugar groups compared to the solid high-sugar groups. However, hepatic fat was up-regulated to some extent in all mice that were fed high-sugar diets (Figure 3D).

### Intestinal sugar transporter expression in obese compared to normal weight humans

**Figure 4:**
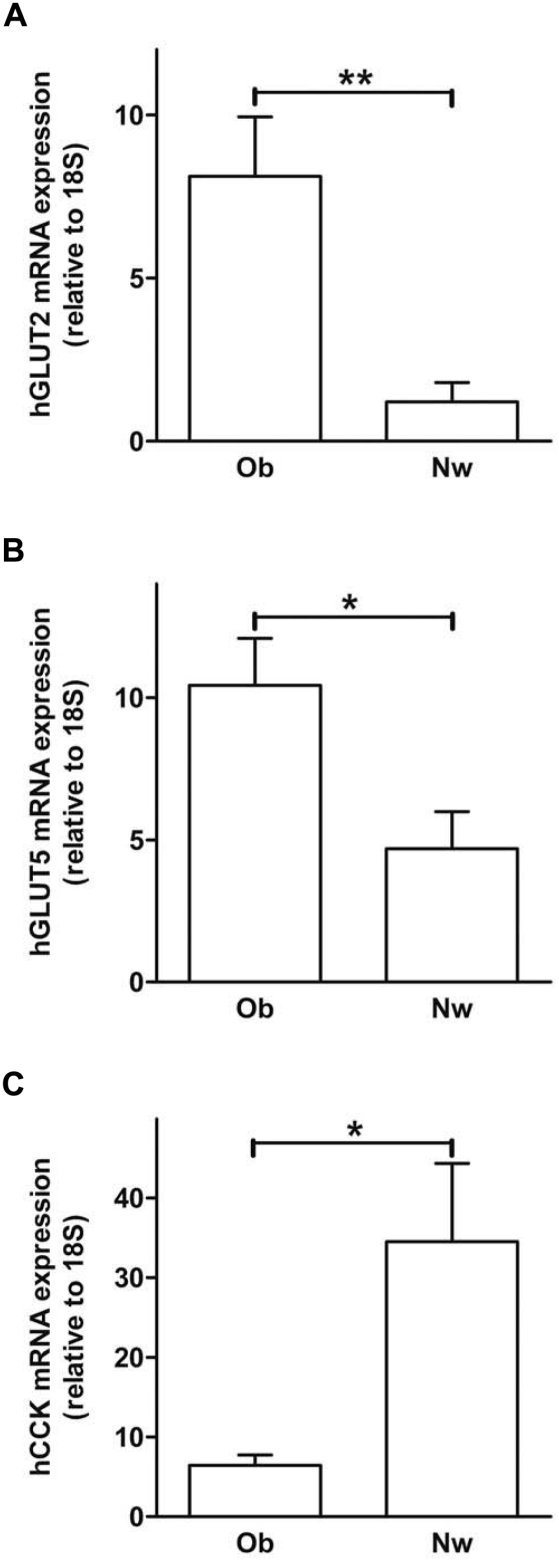
Obese humans showed an increased small intestinal sugar transporter expression compared to normal weight humans. Small intestinal hGLUT2, hGLUT5 and hCCK mRNA expression was detected (A/B/C). Data are shown as means ± SEM (*P<0.05; **P<0.01; n = 12–20). hGLUT2/5, human glucose transporter 2/5; hCCK, human cholecystokinin; Ob, obese; Nw, normal weight.

We found a significant enhancement of GLUT2 (P<0.01; Figure 4A) and GLUT5 (P<0.05; Figure 4B) mRNA expression in human small intestine from obese compared to lean individuals. In contrast, CCK mRNA was down-regulated in obese versus lean humans (P<0.05; Figure 4C).

## Discussion

The results we present here provide evidence that liquid versus solid high-sugar diets differentially modulate feeding behavior, distinct intestinal sugar transporters and weight regulating hormones. Consequently, liquid high-sugar diets may be a critical component for the development of obesity and fatty liver disease in mice. Interestingly, in obese humans we find similar enhanced sugar transporter regulation within the small intestine as in liquid high-sugar diet fed mice, but opposed weight regulating hormone expression.

In a previous study by our group, mice, when fed a liquid high-fructose diet, compensated elevated caloric intake by reducing food intake [28]. In contrast, feeding a liquid high-glucose or -sucrose diet, caloric intake and weight gain were increased compared to control mice [28], [29]. Possibly, feeding behavior is differently regulated for fructose and glucose intake.

In this study, we investigate, if there is a difference in feeding behavior between mice receiving liquid or solid sugars. Therefore, we explicitly compare the effect of sugar administration in solid versus liquid form. The mice show an obvious preference for the liquid high-sucrose diet in terms of liquid sucrose and total caloric ingestion compared to the solid high-sucrose or control diet. Similar observations have been made in man, since sweetened soft drink ingestion is clearly associated with an increased energy intake in humans [30], [31].

In accordance with the elevated caloric intake in the mice fed liquid sucrose, we determine an up-regulation of the ileal sugar transporters GLUT2 and GLUT5 compared to the control mice or mice fed the sucrose in solid form. Although the duodenum is the first site of sugar absorption, the ileum shows the most pronounced effects, assumingly, due to a fast transport of the sucrose solution within the small intestine of the mice.

In addition, previous studies have proven that GLUT2 is primed in mice receiving long term high-sugar diets, as well as in diabetic rats [32]. Similar to our findings, another study reported that a 30 day high-fructose diet resulted in the permanent presence of GLUT2 in the apical membrane [33]. Furthermore, GLUT2 plays a key role in glucose and fructose detection, thus controlling feeding behavior in mice [16]. GLUT2 is also proposed to regulate sugar intake in humans. For example, individuals with a GLUT2 allelic variant (Thr110Ile) from two separate Canadian populations have a higher daily intake of sugars [34].

In line with our findings, other studies verified that a high-fructose diet increased intestinal GLUT5 or GLUT2 short term expression. Similar results were obtained when rodents were intestinally perfused with increasing fructose concentrations [13], [18]. We confirm and extend such data by showing enhanced GLUT2, but not GLUT5 expression feeding the liquid high-fructose diet, when compared to the solid high-fructose or the control diets.

Sugars also stimulate the sweet taste receptor type 1 member 3 (T1R3) and gustducin followed by the up-regulation of SGLT1 expression [13], [35]. Since we measure an increase of T1R3 but not SGLT1 mRNA expression we assume that the here shown effects are due to a long term sugar stimulation (Figure S1). We postulate that SGLT1 elevation may occur within a very short time frame after sugar consumption, keeping in mind that the SGLT1 is saturated by a relatively small sugar concentration (30 mMol). Nevertheless, SGLT1 seems to play a role as a glucose sensor involved in the control of apical GLUT2 insertion [32]. If the SGLT1 is involved in the here shown GLUT2 enhancement needs further investigation.

Similar to the up-regulation of intestinal GLUT2 and GLUT5, the satiety hormone CCK is enhanced in the ileum of mice fed with liquid high-sugar diets, in contrast to mice fed with solid high-sugar diets or control diets. CCK is known to suppress carbohydrate intake via the CCK-A receptor [36]. In both, preclinical and clinical studies, CCK decreased food intake by reducing meal size and duration [37], [38]. However, no reduction of 24-h food intake was seen due to compensatory increases of CCK [39]. Similarly, in clinical trials after 24-h continuous CCK infusion, subjects developed tolerance [40]. Hence, the here shown up-regulation of CCK after feeding liquid high-sugar diets to mice, might be compensated by not yet known mechanisms.

As confirmed for CCK, ghrelin is known to influence feeding behavior in the periphery as well as centrally [41]–[43]. According to our data, the different sugar diets have only minor effects on ghrelin and virtually no effects on nesfatin-1 and PYY expression (data not shown). Consequently, a rather selective influence of sugars in liquid form on particular weight regulating hormones, such as CCK, is anticipated.

Hepatic triglycerides show a similar enhancement when being compared to GLUT2 and CCK mRNA expression in the intestine after feeding liquid high-sugar diets compared to control mice. Our data support a study by Sakar et al. who proposed a positive regulatory control loop between a high-fructose diet and intestinal GLUT2/GLUT5 transporters which is at the same time linked to hepatic metabolic functions in rodents [44]. As expected, liver to body weight ratio is increased in all the high-sugar diet fed mice compared to control mice, which is in agreement with the increased overall hepatic lipid accumulation we see in liver tissue.

Similar to mice fed a high-sugar diet, obese subjects have elevated small intestinal GLUT2 and GLUT5 levels in contrast to lean subjects. Of course, we cannot say that sugars lead to the effects we show in obese humans, but we assume that the excessive consumption of a westerns style diet in the obese group might have an influence on the here shown dysregulation of weight regulating parameters. Underlining our statement, it is clearly shown in the literature [45], [46], that obese have a greater energy intake than expenditure. In contrast, lean humans in general have a balanced energy household. Nevertheless, we have to keep in mind that some studies show no increase in overall dietary carbohydrate uptake in overweight/obese compared to lean subjects [47], [48]. Therefore, it is of importance to calculate the absorbed or metabolisable energy meaning the difference between gross energy in consumed food and energy in feces and urine. We here use values from the literature referring to Southgate and Durnin who showed that with bomb calorimetry determined and on the other hand calculated values are in good agreement [49]. Another report using a rodent model showed that Atwater factors predicted metabolisable energy with satisfactory accuracy in purified diets as we used here [50]. However, it is of note, that data available for our article was measured in humans or chicken [24], [51].

Interestingly, small intestinal CCK is down-regulated in obese compared to lean humans. Our finding suggests a CCK dysregulation that might lead to reduced satiety signaling, boosting the development of obesity.

In conclusion, our data indicates that liquid high-sugar diets compared to solid high-sugar diets differentially modulate feeding behavior, as well as intestinal sugar transporters, and hormone expression. Our study implicates a risk for an increased consumption of sucrose sweetened beverages, followed by elevated intestinal energy uptake and the development of fatty liver disease.

According to the data we present here, antagonists of GLUT2 and GLUT5 might be novel pharmacologic targets for modulating feeding behavior and intestinal sugar uptake in obese patients. GLUT2 and GLUT5 inhibitors could prevent from a dramatically increased intestinal sugar uptake, presumably leading to weight reduction. A combination of medication including GLUT2/5 antagonists and the treatment of negative side effects e.g. diarrhea and flatulence might be a possibility for obese individuals to loose weight. However, if GLUT2/5 antagonists are reliable and sustainable drugs against obesity needs further investigation.

## Supporting Information

Figure S1 **Effects of high-sugar diets on intestinal T1R3 and SGLT1 mRNA expression.** Small intestinal T1R3 and SGLT1 mRNA expression was detected (A/B). Data are shown as means ± SEM (*P<0.05; n = 10). T1R3: taste receptor type 1 member 3; SGLT1: sodium-driven sugar co-transporter 1.
(TIF)

Table S1 **Metabolizable energy of diets.** Protein corrected for unoxidized material estimated about 23% of energy lost in urine and feces [27]. The difference of available energy of liquid and solid diets as well as fructose and sucrose is not significant and was calculated using the 2-way ANOVA. MN, macro nutrients, Fl, fructose liquid; Fs, fructose solid; Sl, sucrose liquid; Ss, sucrose solid; CAE, coefficient of available energy; ME, metabolizable energy.
(DOCX)

## References

[1] Malik VS, Willett WC, Hu FB (2013) Global obesity: trends, risk factors and policy implications. Nat Rev Endocrinol 9: 13–27. 23165161 10.1038/nrendo.2012.199

[2] Siervo M, Montagnese C, Mathers JC, Soroka KR, Stephan BC, et al.. (2013) Sugar consumption and global prevalence of obesity and hypertension: an ecological analysis. Public Health Nutr: 1–10. 10.1017/S1368980013000141 PMC10282320 23414749

[3] Ford CN, Slining MM, Popkin BM (2013) Trends in dietary intake among US 2- to 6-year-old children, 1989–2008. J Acad Nutr Diet 113: 35–42. 23260722 10.1016/j.jand.2012.08.022 PMC3531045

[4] Lustig RH, Schmidt LA, Brindis CD (2012) Public health: The toxic truth about sugar. Nature 482: 27–29. 22297952 10.1038/482027a

[5] Must A, Barish EE, Bandini LG (2009) Modifiable risk factors in relation to changes in BMI and fatness: what have we learned from prospective studies of school-aged children? Int J Obes (Lond) 33: 705–715. 19399020 10.1038/ijo.2009.60 PMC3586424

[6] Tappy L, Le KA (2010) Metabolic effects of fructose and the worldwide increase in obesity. Physiol Rev 90: 23–46. 20086073 10.1152/physrev.00019.2009

[7] Melanson KJ, Angelopoulos TJ, Nguyen V, Zukley L, Lowndes J, et al (2008) High-fructose corn syrup, energy intake, and appetite regulation. Am J Clin Nutr 88: 1738S–1744S. 19064539 10.3945/ajcn.2008.25825E

[8] Pereira MA (2006) The possible role of sugar-sweetened beverages in obesity etiology: a review of the evidence. Int J Obes 30: S28–S36.

[9] Rizkalla SW (2010) Health implications of fructose consumption: A review of recent data. Nutr Metab (Lond) 7: 82. 21050460 10.1186/1743-7075-7-82 PMC2991323

[10] Shirazi-Beechey SP, Moran AW, Batchelor DJ, Daly K, Al-Rammahi M (2011) Glucose sensing and signalling; regulation of intestinal glucose transport. Proc Nutr Soc 70: 185–193. 21450125 10.1017/S0029665111000103

[11] Horio N, Jyotaki M, Yoshida R, Sanematsu K, Shigemura N, et al (2010) New frontiers in gut nutrient sensor research: nutrient sensors in the gastrointestinal tract: modulation of sweet taste sensitivity by leptin. J Pharmacol Sci 112: 8–12. 20093782 10.1254/jphs.09r07fm

[12] Woods SC (2004) Gastrointestinal satiety signals I. An overview of gastrointestinal signals that influence food intake. Am J Physiol Gastrointest Liver Physiol 286: G7–13. 14665437 10.1152/ajpgi.00448.2003

[13] Kellett GL, Brot-Laroche E, Mace OJ, Leturque A (2008) Sugar absorption in the intestine: the role of GLUT2. Annu Rev Nutr 28: 35–54. 18393659 10.1146/annurev.nutr.28.061807.155518

[14] Augustin R (2010) The protein family of glucose transport facilitators: It’s not only about glucose after all. IUBMB Life 62: 315–333. 20209635 10.1002/iub.315

[15] Kellett GL, Helliwell PA (2000) The diffusive component of intestinal glucose absorption is mediated by the glucose-induced recruitment of GLUT2 to the brush-border membrane. Biochem J 350 Pt 1: 155–162. PMC1221237 10926839

[16] Stolarczyk E, Guissard C, Michau A, Even PC, Grosfeld A, et al (2010) Detection of extracellular glucose by GLUT2 contributes to hypothalamic control of food intake. Am J Physiol Endocrinol Metab 298: E1078–1087. 20179244 10.1152/ajpendo.00737.2009

[17] Barone S, Fussell SL, Singh AK, Lucas F, Xu J, et al (2009) Slc2a5 (Glut5) is essential for the absorption of fructose in the intestine and generation of fructose-induced hypertension. J Biol Chem 284: 5056–5066. 19091748 10.1074/jbc.M808128200 PMC2643499

[18] Douard V, Ferraris RP (2008) Regulation of the fructose transporter GLUT5 in health and disease. Am J Physiol Endocrinol Metab 295: E227–237. 18398011 10.1152/ajpendo.90245.2008 PMC2652499

[19] Douard V, Ferraris RP (2013) The role of fructose transporters in diseases linked to excessive fructose intake. J Physiol 591: 401–414. 23129794 10.1113/jphysiol.2011.215731 PMC3577529

[20] Bray GA (2000) Afferent signals regulating food intake. Proc Nutr Soc 59: 373–384. 10997653 10.1017/s0029665100000422

[21] de Graaf C, Blom WA, Smeets PA, Stafleu A, Hendriks HF (2004) Biomarkers of satiation and satiety. Am J Clin Nutr 79: 946–961. 15159223 10.1093/ajcn/79.6.946

[22] Dumoulin V, Moro F, Barcelo A, Dakka T, Cuber JC (1998) Peptide YY, glucagon-like peptide-1, and neurotensin responses to luminal factors in the isolated vascularly perfused rat ileum. Endocrinology 139: 3780–3786. 9724030 10.1210/endo.139.9.6202

[23] Weed DL, Althuis MD, Mink PJ (2011) Quality of reviews on sugar-sweetened beverages and health outcomes: a systematic review. Am J Clin Nutr 94: 1340–1347. 21918218 10.3945/ajcn.111.015875 PMC3192479

[24] Flipot P, Rene JB, Brisson GJ (1971) Availability of the amino acids in casein, fish meal, soya protein and zein as measured in the chicken. Canadian Journal of animal science 51: 801–802.

[25] Atwater WO, Bryant AP (1900) Availability and fuel value of food materials. Storrs Station Report for 1899: 73–100.

[26] Atwater WO (1903) The demands of the body for nourishment and dietary standards. Conn (Storrs) Agr Expt Sta (1902–1903): 123–146.

[27] Rubner M (1885) Calorimetrische Untersuchungen. I. Zeitschrift für Biologie 21: 250–334.

[28] Bergheim I, Weber S, Vos M, Kramer S, Volynets V, et al (2008) Antibiotics protect against fructose-induced hepatic lipid accumulation in mice: role of endotoxin. J Hepatol 48: 983–992. 18395289 10.1016/j.jhep.2008.01.035

[29] Haub S, Kanuri G, Volynets V, Brune T, Bischoff SC, et al (2010) Serotonin reuptake transporter (SERT) plays a critical role in the onset of fructose-induced hepatic steatosis in mice. Am J Physiol Gastrointest Liver Physiol 298: G335–344. 19713474 10.1152/ajpgi.00088.2009

[30] Vartanian LR, Schwartz MB, Brownell KD (2007) Effects of soft drink consumption on nutrition and health: a systematic review and meta-analysis. Am J Public Health 97: 667–675. 17329656 10.2105/AJPH.2005.083782 PMC1829363

[31] Striegel-Moore RH, Thompson D, Affenito SG, Franko DL, Obarzanek E, et al (2006) Correlates of beverage intake in adolescent girls: the National Heart, Lung, and Blood Institute Growth and Health Study. J Pediatr 148: 183–187. 16492426 10.1016/j.jpeds.2005.11.025

[32] Kellett GL, Brot-Laroche E (2005) Apical GLUT2: a major pathway of intestinal sugar absorption. Diabetes 54: 3056–3062. 16186415 10.2337/diabetes.54.10.3056

[33] Tobin V, Le Gall M, Fioramonti X, Stolarczyk E, Blazquez AG, et al (2008) Insulin internalizes GLUT2 in the enterocytes of healthy but not insulin-resistant mice. Diabetes 57: 555–562. 18057092 10.2337/db07-0928

[34] Eny KM, Wolever TM, Fontaine-Bisson B, El-Sohemy A (2008) Genetic variant in the glucose transporter type 2 is associated with higher intakes of sugars in two distinct populations. Physiol Genomics 33: 355–360. 18349384 10.1152/physiolgenomics.00148.2007

[35] Dyer J, Daly K, Salmon KS, Arora DK, Kokrashvili Z, et al (2007) Intestinal glucose sensing and regulation of intestinal glucose absorption. Biochem Soc Trans 35: 1191–1194. 17956309 10.1042/BST0351191

[36] Bellissimo N, Anderson GH (2003) Cholecystokinin-A receptors are involved in food intake suppression in rats after intake of all fats and carbohydrates tested. J Nutr 133: 2319–2325. 12840200 10.1093/jn/133.7.2319

[37] Huda MS, Wilding JP, Pinkney JH (2006) Gut peptides and the regulation of appetite. Obes Rev 7: 163–182. 16629873 10.1111/j.1467-789X.2006.00245.x

[38] Gibbs J, Young RC, Smith GP (1973) Cholecystokinin elicits satiety in rats with open gastric fistulas. Nature 245: 323–325. 4586439 10.1038/245323a0

[39] West DB, Fey D, Woods SC (1984) Cholecystokinin persistently suppresses meal size but not food intake in free-feeding rats. Am J Physiol 246: R776–787. 6326618 10.1152/ajpregu.1984.246.5.R776

[40] Crawley JN, Beinfeld MC (1983) Rapid development of tolerance to the behavioural actions of cholecystokinin. Nature 302: 703–706. 6300693 10.1038/302703a0

[41] Schaeffer M, Langlet F, Lafont C, Molino F, Hodson DJ, et al (2013) Rapid sensing of circulating ghrelin by hypothalamic appetite-modifying neurons. Proc Natl Acad Sci U S A 110: 1512–1517. 23297228 10.1073/pnas.1212137110 PMC3557016

[42] Date Y (2012) Ghrelin and the vagus nerve. Methods Enzymol 514: 261–269. 22975058 10.1016/B978-0-12-381272-8.00016-7

[43] Alvarez-Crespo M, Skibicka KP, Farkas I, Molnar CS, Egecioglu E, et al (2012) The amygdala as a neurobiological target for ghrelin in rats: neuroanatomical, electrophysiological and behavioral evidence. PLoS One 7: e46321. 23071554 10.1371/journal.pone.0046321 PMC3468604

[44] Sakar Y, Nazaret C, Letteron P, Ait Omar A, Avenati M, et al (2009) Positive regulatory control loop between gut leptin and intestinal GLUT2/GLUT5 transporters links to hepatic metabolic functions in rodents. PLoS One 4: e7935. 19956534 10.1371/journal.pone.0007935 PMC2780353

[45] Sharma AM, Padwal R (2010) Obesity is a sign - over-eating is a symptom: an aetiological framework for the assessment and management of obesity. Obes Rev 11: 362–370. 19922430 10.1111/j.1467-789X.2009.00689.x

[46] Chaput JP, Perusse L, Despres JP, Tremblay A, Bouchard C (2014) Findings from the Quebec Family Study on the Etiology of Obesity: Genetics and Environmental Highlights. Curr Obes Rep 3: 54–66. 24533236 10.1007/s13679-013-0086-3 PMC3920031

[47] Coelho SB, de Sales RL, Iyer SS, Bressan J, Costa NM, et al (2006) Effects of peanut oil load on energy expenditure, body composition, lipid profile, and appetite in lean and overweight adults. Nutrition 22: 585–592. 16704951 10.1016/j.nut.2006.03.012

[48] Daousi C, Wilding JP, Aditya S, Durham BH, Cleator J, et al (2009) Effects of peripheral administration of synthetic human glucose-dependent insulinotropic peptide (GIP) on energy expenditure and subjective appetite sensations in healthy normal weight subjects and obese patients with type 2 diabetes. Clin Endocrinol (Oxf) 71: 195–201. 19178509 10.1111/j.1365-2265.2008.03451.x

[49] Southgate DA, Durnin JV (1970) Calorie conversion factors. An experimental reassessment of the factors used in the calculation of the energy value of human diets. Br J Nutr 24: 517–535. 5452702 10.1079/bjn19700050

[50] Bielohuby M, Bodendorf K, Brandstetter H, Bidlingmaier M, Kienzle E (2010) Predicting metabolisable energy in commercial rat diets: physiological fuel values may be misleading. Br J Nutr 103: 1525–1533. 20047701 10.1017/S000711450999345X

[51] Holmes AD (1918) Digestibility of some seed oils. United States Department of Agriculture Bulletin No. 687: 1–24.

