# Supplementary figures and images for: Effect of High Sugar Intake on Glucose Transporter and Weight Regulating Hormones in Mice and Humans
Source: PLoS One. 2014 Jul 10;9(7):e101702. doi: 10.1371/journal.pone.0101702 (PMC4092057; doi:10.1371/journal.pone.0101702)

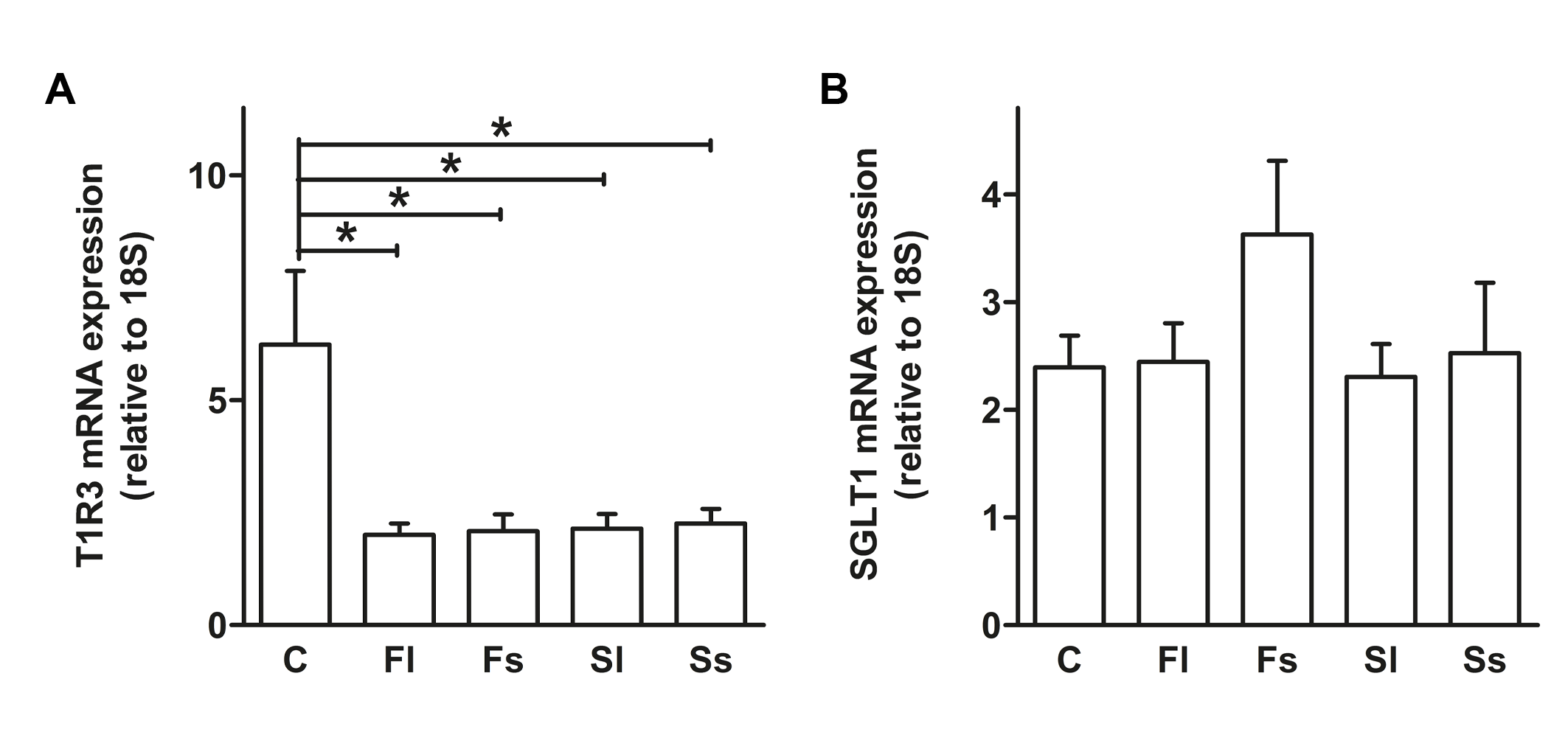

Supplement: Figure S1 — Effects of high-sugar diets on intestinal T1R3 and SGLT1 mRNA expression. Small intestinal T1R3 and SGLT1 mRNA expression was detected (A/B). Data are shown as means ± SEM (*P<0.05; n = 10). T1R3: taste receptor type 1 member 3; SGLT1: sodium-driven sugar co-transporter 1. (TIF) [file pone.0101702.s001.tif]
