# Supplementary material for: Effect of High Sugar Intake on Glucose Transporter and Weight Regulating Hormones in Mice and Humans
Source: PLoS One. 2014 Jul 10;9(7):e101702. doi: 10.1371/journal.pone.0101702 (PMC4092057; doi:10.1371/journal.pone.0101702)
Supplement: Table S1 — Metabolizable energy of diets. Protein corrected for unoxidized material estimated about 23% of energy lost in urine and feces [27]. The difference of available energy of liquid and solid diets as well as fructose and sucrose is not significant and was calculated using the 2-way ANOVA. MN, macro nutrients, Fl, fructose liquid; Fs, fructose solid; Sl, sucrose liquid; Ss, sucrose solid; CAE, coefficient of available energy; ME, metabolizable energy. (DOCX) [file pone.0101702.s002.docx]

**Table S1. Metabolizable energy of diets**

| **MN** |  | **Fl** |  |  | **Fs** |  |  | **Sl** |  |  | **Ss** |  |
| --- | --- | --- | --- | --- | --- | --- | --- | --- | --- | --- | --- | --- |
|  | **Kcal/g/d** | **CAE** | **ME** | **Kcal/g/d** | **CAE** | **ME** | **Kcal/g/d** | **CAE** | **ME** | **Kcal/g/d** | **CAE** | **ME** |
| **Casein** | 1.03 | 96.0% | 0.75 | 1.86 | 96.0% | 1.36 | 0.81 | 96.0% | 0.59 | 1.65 | 96.0% | 1.21 |
| **Sunflower oil** | 0.26 | 96.5% | 0.25 | 0.46 | 96.5% | 0.45 | 0.20 | 96.5% | 0.19 | 0.41 | 96.5% | 0.40 |
| **Corn starch** | 2.72 | 85.0% | 2.31 |  |  |  | 2.14 | 85.0% | 1.82 |  |  |  |
| **Solid sucrose** | 0.51 | 97.0% |  |  |  |  |  |  |  |  |  |  |
| **Solid fructose** |  |  |  | 6.03 | 97.0% | 5.85 | 0.40 | 97.0% | 0.39 | 5.38 | 97.0% | 5.21 |
| **Liquid fructose** | 3.26 | 97.0% | 0.50 |  |  |  | 4.88 | 97.0% |  |  |  |  |
| **Liquid sucrose** |  |  | 3.16 |  |  |  |  |  | 4.74 |  |  |  |
| **Total** |  |  | **6.22** |  |  | **7.66** |  |  | **7.14** |  |  | **6.82** |

Protein corrected for unoxidized material estimated about 23% of energy lost in urine and feces [[27](#_ENREF_27)]. The difference of available energy of liquid and solid diets as well as fructose and sucrose is not significant and was calculated using the 2-way ANOVA. MN, macro nutrients, Fl, fructose liquid; Fs, fructose solid; Sl, sucrose liquid; Ss, sucrose solid; CAE, coefficient of available energy; ME, metabolizable energy.
